# Prevalence and factors associated with overweight and obesity among adults in Hawassa city, southern Ethiopia: a community based cross-sectional study

**DOI:** 10.1186/s40608-019-0227-7

**Published:** 2019-03-04

**Authors:** Teshale Darebo, Addisalem Mesfin, Samson Gebremedhin

**Affiliations:** 1 Department of Public Health, Mizan Tepi University, Mizan Aman, Ethiopia; 2 School of Nutrition, Food Science and Technology, Hawassa University, Hawassa, Ethiopia; 3 School of Public Health, Hawassa University, Hawassa, Ethiopia

**Keywords:** Overweight and obesity, Physical exercise, Body mass index, Risk factors, Hawassa, Ethiopia

## Abstract

**Background:**

In Ethiopia, limited information is available about the epidemiology of over-nutrition. This study assessed the prevalence of, and factors associated with overweight and obesity among adults in Hawassa city, Southern Ethiopia.

**Methods:**

A community-based cross-sectional survey was conducted in August 2015 in the city. A total of 531 adults 18–64 years of age were selected using multistage sampling approach. Interviewer administered qualitative food frequency questionnaire was used to assess the consumption pattern of twelve food groups. The level of physical exercise was measured via the General Physical Activity Questionnaire (GPAQ). Based on anthropometric measurements, Body Mass Index (BMI) was computed and overweight including obesity (BMI of 25 or above) was defined. For identifying predictors of overweight and obesity, multivariable binary logistic regression model was fitted and the outputs are presented using Adjusted Odds Ratio (AOR) with 95% Confidence Intervals (CI).

**Results:**

The prevalence of overweight including obesity was 28.2% (95% CI: 24.2–32.2). Significant proportions of adults had moderate (37.6%) or low (2.6%) physical activity level. As compared to men, women had 2.56 (95% CI: 1.85–4.76) times increased odds of overweight/obesity. With reference to adults 18–24 years of age, the odds were three times higher among adults 45–54 (3.06, 95% CI: 1.29–7.20) and 55–64 (2.88, 95% CI: 1.06–7.84) years. Those from the highest income tercile were 3.16 times (95% CI: 1.88–5.30) more likely to be overweight/obese as compared to adults from the lowest tercile. Having moderate (3.10, 95% CI: 1.72–5.60) or low (4.80 95% CI: 2.50–9.23) physical activity was also significantly associated with the outcome. Further, daily intake of alcohol and, frequent consumption of sweets, meat and eggs were associated with overweight/obesity. Conversely, no significant associations were evident for meal frequency, practices of skipping breakfast, behavior of eating away from home and frequency of consumption of fast foods, fruits and vegetables.

**Conclusions:**

Prompting active lifestyle, limiting intakes of sweets, advocating optimum consumption of alcohol and calorie dense animal source foods, especially amongst the better-off segment of the population, may reduce the magnitude of over-nutrition.

**Electronic supplementary material:**

The online version of this article (10.1186/s40608-019-0227-7) contains supplementary material, which is available to authorized users.

## Background

Globally, each year non-communicable diseases (NCDs) cause 40 million deaths and loss of 1.5 million disability-adjusted life years (DALY) [1, 2]. Cardiovascular diseases, cancers, chronic respiratory disease and diabetes mellitus in combination cause more than three-fourth of all global deaths secondary to NCDs. In absolute numbers, the four medical conditions alone annually result in 32 million deaths throughout the world [1]. In the last two decades, the epidemiological significance of NCDs has been rapidly growing both in developed and developing countries. Globally between 2000 and 2015, the total adult mortality attributable to NCDs has increased by 16% [3].

Non-communicable diseases have wide-range of genetic, environmental and behavioral determinants. However, the rapid epidemiological shift observed in the last few decades is largely attributable to changes in few, known and modifiable risk factors. These factors are tobacco and alcohol use, physical inactivity, unhealthy diets, raised blood pressure, overweight/obesity, hyperglycemia and hyperlipidemia [4]. Most of the risk factors function in complex and synergistic fashion and, tend to co-occur together [2, 4].

Overweight including obesity refer to abnormal or excessive accumulation of fat that may result in health impairment and its usually measured based on high Body Mass Index (BMI) [1]. There is convincing evidence that overweight and obesity increase the risk of several types of cancers and elevate blood pressure, cholesterol and insulin resistance [4]. Each year worldwide, 2.8 million deaths and 2.3% of the global DALY loss are attributed to the consequences of overweight or obesity [5]. In the last few decades, a substantial rise in the prevalence of overweight and obesity has been observed both in developed and developing countries [6, 7]. According to the World Health Organization (WHO), in 2014 globally approximately two billion adults – 39% of women and men – were overweight or obese [7, 8]. Between 1975 and 2016, the combined prevalence almost doubled from the baseline level of 22% [7].

According to the estimate of WHO, in 2014 1.2% of men and 6.0% of females were either overweight or obese in Ethiopia [7]. Between 1997 and 2016, the combined prevalence in the country grew considerably from 2.6 to 6.9% in females and, from 0.6 to 1.9% in males [7]. Presumably, in urban areas of Ethiopia including Hawassa city, the prevalence is growing. Yet, epidemiological evidence on the underlying risk factors is scarce. The recent Ethiopian Demographic and Health Survey (DHS) determined the national magnitude of overweight and obesity both in urban and rural areas. However, limited information is provided on the underlying drivers of the problem. While the general risk factors for overweight and obesity are known; the magnitude and strength of association of the factors, and hence their practical significance, may vary from one setting to another. Hence this study was conducted to assess the prevalence of, and factors associated with overweight and obesity among adult (18–64 years) in Hawassa city, Southern Ethiopia.

### Study setting

Hawassa is the capital city of Southern Nations Nationalities Peoples Region (SNNPR), located 273 km south of Addis Ababa, the capital of Ethiopia. Projection based on the 2007 national census indicates, as of 2015 the city had a total population of 319,023, of whom 51.9% were adults18–64 years of age [9].

### Study design and study participants

This community-based cross-sectional study was conducted in 2015. Adults 18 years of age or older who were the permanent residents of Hawassa city were eligible for the study. Pregnant women, women in the first 6 months after giving birth and adults with obvious spinal deformity were excluded from the study.

### Sample size

The sample size for the study was determined using single population proportion formula. The formula estimates the minimum sample size required for determining a proportion in a source population as a function of desired level of significance, margin of error and the expected level of the proportion. Specifications made during the computation were: 21% expected prevalence of overweight and obesity [10], 95% confidence level, 5% margin of error, 10% compensation for possible non-response and design effect of 2. The final calculated sample size was 565. The adequacy of the sample size for determining the factors associated with overweight and obesity was assessed using a post-hoc power calculation.

### Sampling technique

Study participants were selected using multistage cluster sampling technique. Initially, 6 kebeles (the smallest administrative unit in Ethiopia) were randomly selected out of the existing 32 kebeles in the city, and the total sample size was distributed to the selected kebeles proportional to their population size. Then, using existing administrative records as sampling frame, households were selected via systematic random sampling approach. From each of the selected household, an adult aged 18–64 years was drawn. When multiple adults were available within the household, an adult was chosen at random from the same household. Conversely, when no adult was available, one was selected from the immediate neighboring household. If sampled adult was absent from their home on the data collection day, another visit was arranged.

### Data collection

Data were collected by trained data collectors and supervisors using structured and pretested questionnaire. The questionnaire was prepared in English, and finalized and administered in Amharic language which is widely spoken in the city. Questions pertaining to socio-demographic characteristics were directly adopted from the standard Demographic and Health Survey (DHS) questionnaire [10]. On average, each interview lasts for 40 min.

The frequency of consumption of twelve food groups was assessed using a qualitative food frequency questionnaire (FFQ) over the reference period of 1 month. As we could not get a validated FFQ, we developed and used our own tool. The twelve food groups were: cereals, roots and tubers, legumes, fruits, vegetables, eggs, milk and milk products, meat, fish, oil and fat, sweets and sugar, and coffee/tea. For each of the 12 food groups, the pattern of consumption was ultimately classified into six ordinal categories: more than once per day, once per day, 3–6 times per week, 1–2 times per week, 1–2 times per month and less than once per month.

The level of physical activity was measured using the Global Physical Activity Questionnaire (GPAQ) [11]. The questionnaire collects information on the level of physical activity in three domains: (1) at work; (2) during transport; and 3) at leisure/recreational activity).ultimately each individual was classified as having low, moderate or high level of physical activity following a standard classification approach [11]. Operationally, low physical activity is a sedentary activity level which describes someone who gets little to no exercise; moderate physical activity is a low-impact aerobic exercise classes, brisk walking or hiking, recreational team sports (volleyball, soccer, etc.); and vigorous physical activity level is running or jogging, high-intensity aerobic classes or competitive full-field sports.

Anthropometric measurements (height and weight) were taken by using calibrated equipment and standardized techniques. For minimizing possible errors, measurements were made in duplicate by the same observer. Weight was measured using SECA electronic weighing scales to the nearest 100 g. The scale was calibrated regularly and the indicator was checked against zero reading prior to every measurement. Height was measured to the nearest 0.1 cm using a standing height board. Ultimately, BMI (wt/ht^2^) was calculated and overweight (BMI of ≥25 to < 30), and obesity (BMI of 30 or above) were classified based on standard thresholds.

### Variables of the study

The dependent variable of the study was presence or absence of overweight or obesity. On the other hand 27 independent variables were considered for the study. These were: (1) age, (2) gender, (3) educational status, (4) type of occupation, (5) household income terciles (low, middle, high), (6) ethnicity, (7) religion, (8) marital status, (9) family size, (10) awareness about chronic diseases, (11) level of physical activity, (12) behavior of skipping breakfast, (13) frequency of consumption away from home, (14) frequency of consumption of fast foods, (15) frequency of alcohol intake, and consumption pattern of the 12 food groups listed earlier. Consequently, the frequencies of consumption of the twelve food groups were treated as twelve different variables. Information on how the variables were measured and categorized is provided as a (Additional file 1).

### Data analysis

Data were analyzed using SPSS version 20. Data were described using frequency distributions, measures of central tendency and dispersion. Factors association with overweight and obesity were identified using bivariable and multivariable binary logistic regression analyses and the findings are presented using both crude odds ratio (COR) and adjusted odds ratio (AOR).

The procedure of developing the multivariable model was as follows: at the beginning, multiple bivariable logistic regression analyses were run for each of the independent variables and then variables that had *p*-value less than 0.25 were considered candidate variables for the multivariable model. In the final multivariable model,α value of 0.05 for two-sided tests was set as the level of significance. Model fitness was assessed using the Hosmer-Lemeshow statistic. In the final model, extent of multicollinearity was measured using variance inflation factor (VIF) which was found to be within a tolerable range (less than 10).

### Ethical consideration

The study was cleared by the Institutional Review Board (IRB) of College of Medicine and Health Sciences, Hawassa University. Data were collected after taking informed written consent from the respondents. All the information gathered was kept confidential and all overweight or obese adults were provided a brief nutrition education.

## Results

### Socio-demographic and economic characteristics

Among 565 adults approached for the study, 524 were willing to take part making the response rate 92.7%. The mean (± SD) age of the respondents was 31.0 (±7.5) years and the majorities (72.7%) were below the age of 35 years. Gender-wise, females were slightly over represented (56.1%) than males (43.9%). About a quarter (25.4%) of the respondents had tertiary level of education and 27.1% had secondary level of education. Approximately half (55.1%) of the respondents were married and 48.5% were living with two or more family members. Above one-third (40.1%) of the respondents had monthly income between 45 and 82 USD. Among the study participants, Sidama and Orthodox Christianity were the leading ethnic group and religious affiliation, respectively (Table 1).

**Table 1:** Socio-demographic characteristics of the study participants, Hawassa city, Southern Ethiopia, June 2015

Variables (*n* = 524)	Frequency	Percent
Sex		
Male	230	43.9
Female	294	56.1
Age (years)		
18–24	194	37.0
25–34	187	35.7
35–44	75	14.0
45–54	42	8.3
55–64	26	5.0
Religion		
Orthodox	213	40.6
Protestant	201	38.4
Catholic	56	10.7
Muslim	44	8.4
Other	12	2.3
Ethnicity		
Sidama	178	34.0
Wolayita	99	19.1
Amahra	73	14.1
Oromo	52	10.2
Tigre	41	8.0
Kambata	40	6.1
Gurage	30	5.7
Others	19	3.6
Marital status		
Married	288	55.1
Single	215	41.3
Divorced	11	2.1
Widowed/Widower	10	1.9
Educational status		
No formal education	36	6.8
1–4 grade	77	14.7
5–8 grade	136	25.9
9–12 grade	142	27.1
Tertiary education	133	25.4
Family size		
1–2	254	48.5
2–4	169	32.3
4–12	101	19.2
Household monthly income (USD)		
< 45	187	35.7
45–82	228	43.5
> 82	109	20.8
Occupation		
Government	122	23.3
Daily laborer	111	21.2
Self-employed	102	19.5
Non-governmental employee	88	16.8
Housewife	82	15.6
Others	19	3.6

### Prevalence of physical inactivity

As described earlier the total physical activity level of respondents was assessed and classified using the General Physical Activity Questionnaires (GPAQ). It was found that 37.6 and 40.8% of the adults were involved in moderate and high physical activity levels respectively; whereas, 21.6% had sedentary life style.

### Selected dietary practices

The study explored the typical daily meal frequency, practices of skipping breakfast, behavior of eating away from home and, frequencies of consumption of fast foods and alcohol. The majorities (60.4%) typically eat three meals a day and 54.4% take an additional snack every day. The practice of skipping breakfast appears to be common. Nearly half (50.2%) mentioned that they omit breakfast at least once per a typical week. About a third of the participants (34.7%) reported frequent (2–6 times per week) eating away from home; whereas 27.7% consume fast foods once per day. More than a quarter (28.9%) took alcohol at least once in the previous month; of them, 30.9% took alcohol on daily basis (Table 2).

**Table 2:** Selected dietary practices of adults 18–64 years of age, Hawassa city, Southern Ethiopia, June 2015

Variables (n = 524)	Frequency	Percentage
Typical meal frequency per day		
Two or less	132	25.2
Three	317	60.4
Four or more	75	14.3
Typical snack frequency per day		
Never	172	32.8
Once	285	54.4
Two or more	67	12.8
Practice of skipping breakfast/week		
Never	261	49.8
One to three	212	40.4
Four to six	34	6.5
Everyday	17	3.3
Consumption of meal away from home per week		
Never	212	40.4
One	130	24.9
Two to six	182	34.7
Frequency of consumption of fast foods		
Once per day	145	27.7
3 to 5 times/week	182	34.7
1–2 times/week	66	12.6
1–2 times/month	62	11.9
0 times/month	68	13.9
Alcohol intake in the last one month		
Yes	152	28.9
No	372	71.1
Alcohol intake frequency within the last one month (*n* = 152)		
Daily	47	30.9
1–6 times per week	41	27.1
Less than once per week	64	42.0

### Frequency of consumption of various food groups

The pattern of consumption twelve food groups was assessed using a qualitative food frequency questionnaire in terms of the number of times per month the food groups were consumed. It was found that all of the respondents daily consume cereals and foods made of oil, butter or fat. Frequencies of legumes (65.3%) and, roots and tubers (37.8%) intake at least once per day were also reasonably high. Proportions that reported of consumption of the following food groups on daily bases on decreasing order were: dairy product (33.6%), fruits (31.3%), meat (28.6%), vegetables (22.3%) and fish (9.7%). Coffee and tea are regularly consumed in the city, almost a quarter (23.3%) take the beverages more than once per day. Further, 21.5% consume sweets foods and beverages on daily basis (Table 3).

**Table 3:** Frequency of consumption of twelve food groups among adults 18–64 years of age, Hawassa city, Ethiopia, 2015

Food groups	%					
	> once/day	Once/day	3–6 times/wk	1–2 times/wk	1–2 times/mo	< once/mo
Cereals	3.6	96.4	0.0	0.0	0.0	0.0
Oil and fat	14.9	85.1	0.0	0.0	0.0	0.0
Legumes	1.2	64.1	16.2	9.9	5.3	3.1
Roots and tubers	6.3	37.8	27.1	24.2	4.6	0.0
Dairy	0.0	33.6	22.1	20.1	13.0	11.1
Fruits	0.0	31.3	35.1	33.6	0.0	0.0
Meat	15.8	28.6	17.7	18.7	16.6	2.5
Vegetables	0.0	22.3	18.3	27.9	31.5	0.0
Fish	0.0	9.7	9.7	10.5	36.5	33.6
Eggs	0.0	21.4	21.6	28.1	29.0	0.0
Sweets and sugar	0.0	21.5	27.4	26.9	24.0	0.0
Coffee and tea	23.3	16.8	23.7	20.4	13.9	1.9

### Prevalence of overweight and obesity

The mean (±SD) BMI of the respondents was 23.7 (±4.1) kg/m^2^. The survey indicated 28.2% (95% CI: 24.2–32.2%) of adults in Hawassa had a BMI of more than 25 kg/m^2^ and were thus overweight or obese. Twenty three percent of the adults were overweight and 5 % were obese. The total prevalence of overweight and obesity in women (36.4%) was more than two times higher than that of the prevalence in men (17.8%). The prevalence of overweight, including obesity rises from 24.2% in adults 18–24 years to, 49.8 and 54.2% among adults 45–54 and 55–64 years of age, respectively.

### Factors associated with overweight and obesity

As described in the variables of the study sub-section, 27 independent variables have been considered for the bivariable logistic regression analyses. Ten independent variables that showed *p*-value less than 0.25 in bivariable analyses were considered as candidate variables for the multivariable model. In multivariable logistic regression analysis, sex, age, physical activity level, frequency of alcohol consumption and frequency of consumption of meat, eggs, and sweet foods and household income were significantly associated (*p* < 0.05) with overweight including obesity.

Based on the adjusted odds ratio, it was found that women, as compared to men, had 2.56 (95% CI: 1.85–4.76) times increased odds of overweight/obesity. With reference to adults 18–24 years old, the odds were three times raised among adults 45–54 (3.06, 95% CI: 1.29–7.20) and 55–64 (2.88, 95% CI: 1.06–7.84) years of age. Similarly, adults from the highest income tercile were 3.16 times (95% CI: 1.88–5.30) more likely to be overweight/obesity that those from the lowest tercile. As compared to adults having high physical activity, those with moderate (3.10, 95% CI: 1.72–5.60) or low (4.80 95% CI: 2.50–9.23) physical activity demonstrated significantly increased odds of overweight/obesity. Further, daily consumptions of alcohol and more frequent intakes of sweets, meat and eggs all showed significant association with overweight/obesity (Table 4).

**Table 4:** Factors association with overweight and obesity among adults 18–64 years of age, Hawassa city, Ethiopia, 2015

Independent variables	Overweight/obesity		Odds ratio (95% CI)	
	Yes	No	Crude (COR)	Adjusted (AOR)
Sex				
Male	41	189	1^r^	1^r^
Female	107	187	2.63 (1.72–4.00)^a^	2.56 (1.85–4.76)^a^
Age (years)				
18–24	47	147	1^r^	1^r^
25–34	20	55	1.14 (0.62–2.08)	1.30 (0.74–2.29)
35–44	50	137	1.14 (0.72–1.81)	1.28 (0.61–2.68)
45–54	18	25	2.25 (1.13–4.49)^a^	3.06 (1.29–7.20)^a^
55–64	13	11	3.69 (1.55–8.80)^a^	2.88 (1.06–7.84)^a^
Physical activity level				
High	35	179	1^r^	1^r^
Moderate	66	131	2.57 (1.61–4.11)^a^	3.10 (1.72–5.60)^a^
Low	47	66	3.62 (2.16–6.13)^a^	4.80 (2.50–9.23)^a^
Alcohols intake last month				
Daily	57	105	1.89 (1.21–2.98)^a^	2.54 (1.40–4.60)^a^
Weekly	42	100	1.47 (0.91–2.37)	1.80 (0.95–3.14)
Never	49	171	1^r^	1^r^
Frequency of consumption of vegetables				
More than once per day	22	82	0.54 (0.30–0.95)^a^	0.58 (0.30–1.10)
Once per day	38	103	0.74 (0.45–1.21)	0.76 (0.38–1.51)
3–6 times per week	33	81	0.81 (0.49–1.37)	0.76 (0.41–1.43)
1–2 times per week	55	110	1^r^	1^r^
Frequency of consumption of fruits				
Once per day	51	113	0.97 (0.61–1.53)	1.16 (0.64–2.13)
3–6 times per week	41	143	0.61 (0.38–0.98)^a^	0.71 (0.39–1.29)
1–2 times per week	56	120	1^r^	1^r^
Frequency of consumption of meat				
More than once per day	37	46	7.23 (3.33–15.85)	4.34 (1.95–9.64)^a^
Once per day	48	102	4.23 (2.03–8.86)^a^	3.05 (1.44–6.46)^a^
3–6 times per week	27	66	3.68 (1.67–8.13)^a^	2.01 (0.87–4.66)
1–2 times per week	26	72	3.25 (1.47–7.18)^a^	1.76 (0.75–4.22)
2 or less times per month	10	90	1^r^	1^r^
Frequency of consumption of egg				
Once per day	49	63	2.64 (1.56–4.46)^a^	2.15 (1.25–3.71)^a^
3–6 times per week	33	80	1.40 (0.81–2.42)	0.97 (0.54–1.77)
1–2 times per week	36	111	1.10 (0.65–1.86)	0.79 (0.45–1.40)
2 or less times per month	30	122	1^r^	1^r^
Frequency of consumption of sweets				
Once per day	51	62	6.09 (3.16–11.70)^a^	4.77 (2.18–10.43)^a^
3–6 times per week	46	98	3.47 (1.83–6.61)^a^	3.47 (1.63–7.38)^a^
1–2 times per week	36	105	2.54 (1.32–5.01)^a^	3.07 (1.42–6.63)^a^
2 or less times per month	15	111	1^r^	1^r^
Household income level				
Low	39	148	1^r^	1^r^
Medium	59	169	1.32 (0.84–2.10)	1.32 (0.83–2.10)
High	50	59	3.22 (1.92–5.39)^a^	3.16 (1.88–5.30)^a^

^a^Significant association at 95% confidence level, 1^r^ base group

No significant associations were evident for the other variables including meal frequency, practices of skipping breakfast, behavior of eating away from home, frequency of consumption of fast foods and pattern of consumption of fruits and vegetables.

## Discussion

The study found substantial differences in the prevalence of overweight and obesity between women (36%) and men (18%). The recent national DHS survey conducted in 2016 similarly reported that in the urban areas of Ethiopia 21% of women and 12% of men; and in Addis Ababa city 30% of women and 20% of men, were either overweight or obese [12]. Studies conducted in many developing countries concluded the same [13–16].The observed difference between the two genders can be due to both biological and social factors. In developing countries males are more frequently engage in physically demanding activities than women do, hence they may have reduced risk of obesity. Further, studies suggested female sex hormones have a great impact on deposition of fat, and hence risk of obesity [17, 18].

The study suggested that the prevalence of overweight and obesity increases with age. The Ethiopia DHS also documented a similar pattern of association, both among men and women [12]. As compared to adults 20–29 years of age, the prevalence of overweight and obesity was almost double among those 40–49 years of age [12]. Many studies conducted in developed and developing countries indicated that mean body weight and BMI gradually increase during late adulthood life [19–23]. The association is likely to be due to natural changes in body composition and decreasing rate of metabolism associated with aging [23].

In Ethiopia very few studies attempted to measure the level physical activity in adults and other segments of the population [24, 25]. Using the GPAQ, our study suggested that sedentary life style (21.6%) is common among adults in Hawassa city and physical inactivity was significantly association with overweight and obesity. A study conducted in Addis Ababa city, Ethiopia also concluded that about two-thirds (65.9%) of working adults in the city had moderate or low physical activity level; furthermore, sedentary life style was inversely associated with central obesity [24].

The study observed a positive relationship between income terciles and prevalence of overweight and obesity. Previous studies so far suggested two divergent patterns of relationship between wealth and overweight/obesity status [26]. Studies from developed countries often suggested that poverty is associated with higher risks for obesity because the economically disadvantaged people are more likely to consume junk and empty calorie foods which are important risk factors of obesity [26, 27]. On the other hand, studies from developing countries frequently documented that obesity increases with wealth because, in transition societies overeating is primarily explained by economic access to food [26, 28, 29]. Parallel to our findings, the Ethiopia DHS indicated the magnitude of overweight including obesity is 8–11 times higher among women and men from households with the highest wealth quintile as compared to those from the lowest quintile [12].

We observed that the odds overweight and obesity are 2.5 times higher among adults who reported daily consumption of alcohol as compared to those who did not take alcohol in the past month. Yet, those who reported weekly alcohol intake demonstrated no increased odds overweight/obesity. Likewise, a recent systematic review concluded that light-to-moderate alcohol intake is not associated with obesity while heavy drinking is more consistently related to weight gain [30]. A secondary analysis of data from the third National Health and Nutrition Examination Survey of the United States also observed more or less similar pattern of association [31]. Alcohol is an important source of condensed calorie and positive energy balance. Further, it may hamper lipid oxidation and increase appetite to foster adiposity [32–34].

We found that odds of overweight including obesity increases with rise in frequency of consumption of sweet food and beverages, meat and eggs. This may indicate that the three food groups are important sources of positive energy balance among adults in the city. Many epidemiological studies testified that regular consumption of sweets and calorie dense animal source foods and are important causes of weight gain. A wealth of evidence shows excessive consumption of sugar and sweets is the root of the global pandemic of obesity and metabolic syndrome [35, 36]. Likewise, a recent ecological study based on data obtained from 170 countries suggested, the consumption of meat contributes just as much as sugar to the growing burden obesity [37]. However, the contribution of frequent consumption of eggs for obesity is less studied and inconsistently observed [38–40].

The study identified multiple factors that are significantly associated with overweight including obesity. However, all of the variables may not have practical significance as some of the predictors (age and sex) are not modifiable. Furthermore, inconsideration of their prevalence and strength of association with the outcome, all the modifiable factors may not have equal practical significance. Based on our findings we recommend that increasing the physical activity level and reducing consumption of animal source foods especially meat, might be the most important interventions to reduce the burden of overweight and obesity in the city.

The major strength of the study is the fact that we enrolled relatively large number of subjects using random sampling approach. This is likely to assure the representativeness of the findings of study to the entire city. Further, the community-based nature of the study contributes to the external validity of the study. We have also attempted to measure multiple variables that having significance for overweight/obesity including level of physical activity.

However, the findings of the study should be interpreted in consideration of the following methodological shortcomings. Some of the independent variables including frequencies of consumption of various food groups, fast foods, alcohol and eating away from home are liable to recall error and this might partially obscure their association with overweight/obesity. Further, as witnessed in many dietary surveys, some individuals may intentionally under-report high dietary intake and over- report low intake to simulate healthy diet; accordingly, the food frequency assessment might have been affected by social desirability bias. While assessing the alcohol intake, we only measured the frequency of consumption without considering the content and volume of alcohol intake. This might have resulted in underestimation of the strength of association with the outcome. We assessed the consumption frequency of different food groups based on a FFQ that has not been validated before, so this might have affected validity of the measurement. As many observational studies, residual confounding from unmeasured or misclassified variables cannot be excluded and, due to the cross-sectional nature of the study, the reported associations may not directly imply causality.

## Conclusion

The study showed that more than a quarter adults in Hawassa city are either overweight or obese. Substantial proportions have moderate (37.6%) or low (21.6%) physical activity levels and such adults have increased likelihood of overweight/obesity. Significantly increased odds of overweight/obesity are also observed among women, adults 45–64 years of age and adults from the highest income tercile. Further, regular alcohol users, and frequent consumers of sweets, meat and eggs had increased odds of the problem.

Promotion of active lifestyle, creating an enabling environment for voluntary physical exercise, limiting intake of sweets, advocating optimum consumption of alcohol and nutrient rich animal source foods especially amongst the better-off segment of the population may help to reduce the magnitude of overweight and obesity.

## Additional file

Additional file 1: Variables. (DOCX 21 kb)

## Abbreviations

AOR: Adjusted Odds Ratio
BMI: Body Mass Index
CI: Confidence Interval
COR: Crude Odds Ratio
DALY: Disability Adjusted Life Years
EDHS: Ethiopian Demographic and Heath Survey
FFQ: Food Frequency Questionnaire
GPAQ: Global Physical Activity Questionnaire
IRB: Institutional Review Board
NCD: Non-communicable Diseases
SD: Standard deviation
SNNPR: Southern Nation Nationality and Peoples Region
SPSS: Statistical Package of Social Science
VIF: Variance Inflation Factor
WHO: World health organization
