# Supplementary material for: Prevalence and factors associated with overweight and obesity among adults in Hawassa city, southern Ethiopia: a community based cross-sectional study
Source: BMC Obes. 2019 Mar 4;6:8. doi: 10.1186/s40608-019-0227-7 (PMC6398229; doi:10.1186/s40608-019-0227-7)
Supplement: Supplementary file 1 — Variables. (DOCX 21 kb) [file 40608_2019_227_MOESM1_ESM.docx]

**Socio-demographic characteristics**

| Questions | Response |
| --- | --- |
| Sex | 1 male  2 female |
| Age | The respondents reported their age in completed years and we categorized it as it is reported in the tables |
| Family size | The respondents reported their family size and we categorized it for the analysis |
| Religion | 1 Orthodox  2 Muslim  3 protestant  4 Catholic  5 Others |
| Ethnicity | We listed the common ethnic groups of the city |
| Marital status | 1 Single  2 Married  3 Divorced  4 Widowed |
| Education completed | 1 illiterate  2 write and read  3 primary school  4 secondary school  5 higher level |
| Average monthly income | The respondents reported their income and we categorized it for the analysis |
| Occupation? | 1 Governmental  2 Non-governmental  3 Self employed  4 Farmer  5 Daily laborer  6 Housewife  7 others |

**Physical activity questionnaire (WHO, 2007)**

| P1 | Do you engaged in any work (including house hold activities) ? | 1 yes  2 no  If 2, go to P8 |
| --- | --- | --- |
| P2 | Does your work involves vigorous-intensity activity that causes large increase in breathing or heart rate like ( carrying or lifting heavy loads, digging or construction work) for at least 10 minutes continuously? | 1 yes  2 No  If 2, go to p5 |
| P3 | In a typical week, on how many days do you do vigorous-intensity activities as part of your work? | --------- day |
| P4 | How much do you spend doing vigorous– intensity activities at work on a typical day? | Hrs : min |
| P5 | Does your work involve moderate-intensity activity that causes small increase in breathing or heart rate such as brisk walking (or carrying light loads) for at least 10 minutes continuously? | 1 yes  2 No  If 2 , go to P 8 |
| P6 | In a typical week, on how many days do you do moderate-intensity activities as part of your work? | ---------- days |
| P7 | How much time do you spend doing moderate-intensity activities at work on a typical day? | Hrs:min--------- |
| P8 | Do you walk or use a bicycle for at least 10 minutes continuously to get to and from places? | 1 yes  2 No  If 2 , go to p11 |
| P9 | In a typical week on how many days do you walk or use bicycle for at least 10 minutes continuously? | ---------- days |
| P10 | How much time do you spend walking or bicycling for travel on a typical day? | -------- days |
| P11 | Do you do any vigorous-intensity sport that causes large increase in breathing or heart rate for at least 10 minutes continuously? | 1 yes  2 No  If 2, go to P14 |
| P12 | In a typical week, on how many days do you do vigorous-intensity sports, fitness or recreational (leisure)activities like lifting heavy loads, football and etc | --------- days |
| P13 | How much time do you spend doing vigorous-intensity sports, fitness and recreational activities (leisure) on a typical day? | Hrs:min-------- |
| P14 | Do you do any moderate-intensity sports, fitness and recreational activities that cause a small increase in breathing or heart rate such as brisk walking, cycling, and volleyball for at least 10 minutes continuously? | 1 yes  2 No  If 2 , go to P17 |
| P15 | In a typical week, on how many days do you do moderate-intensity sports, fitness and recreational activities? | -------- days |
| P16 | How much time do you spend doing moderate-intensity sports, fitness and recreational activities on typical day? | Hrs:min------------ |
| P17 | How much time do you usually spend sitting or reclining on a typical day? | Hrs:min------- |

**Knowledge about chronic diseases**

| Have you ever heard about chronic diseases? | 1 yes  2 No |
| --- | --- |
| If yes, do you know the causes of chronic diseases? | 1 yes  2 No |
| If yes, please mention at least two causes? |  |

**Food frequency**

| S. no | Food groups | >1x/  day | 1x/  day | 3-6x/ week | 1-2x/  week | 1-2x/  Week | Never |
| --- | --- | --- | --- | --- | --- | --- | --- |
| 1 | Any food made from cereals, grains (e.g made of maize, sorghum, millet, wheat, barley, teff) |  |  |  |  |  |  |
| 2 | Any food made from root and tubers (potatoes, sweet potatoes…) |  |  |  |  |  |  |
| 3 | Any vegetable? |  |  |  |  |  |  |
| 4 | Any fruits? |  |  |  |  |  |  |
| 5 | Any beef lamb, goat, chicken, or other meat? |  |  |  |  |  |  |
| 6 | Any eggs? |  |  |  |  |  |  |
| 7 | Any fish? |  |  |  |  |  |  |
| 8 | Any food made from beans (e.g kidney beans, haricot beans, field peas, cow peas, chickpeas or others |  |  |  |  |  |  |
| 9 | Milk and its products? |  |  |  |  |  |  |
| 10 | Any food with oil, fat, or butter? |  |  |  |  |  |  |
| 11 | Any sugar, sweet/soft drinks? |  |  |  |  |  |  |
| 12 | Tea or coffee |  |  |  |  |  |  |

**Selected dietary practices**

| On average, how many meals do you have per a day? |  |
| --- | --- |
| Do you have a habit of having snacks between meals? | 1 yes  2 No |
| If yes, on average how many times per a day? |  |
| Do you have a habit of skipping breakfast? | 1 yes  2 No |
| If yes, on average how many times do you skip your breakfast per a week? |  |
| Do you have a habit of eating away from home? | 1 yes  2 No |
| If yes, on average how many times per week do you eat away from home? |  |
| Do you have a habit of eating fast foods? | 1 yes  2 No |
| If yes, in the last one month, how many times did you eat fast foods | They were further probed to remember by recalling the type of fast foods available in the city |
| Do you have a habit of taking alcohol? | 1 yes  2 No |
| If yes, in the last one month, how many times did you take alcohol |  |
